# Long‐Term Outcomes of Necrotizing Pneumonia and Parapneumonic Effusion in Children

**DOI:** 10.1002/ppul.71241

**Published:** 2025-08-19

**Authors:** Sinem Can Oksay, Begüm Yörük, Şeyda Karabulut, Hüseyin Arslan, Gülay Bilgin, Füsun Ünal, Deniz Mavi Tortop, Ebru Köstereli, Zeynep Reyhan Onay, Yasemin Gokdemir, Zeynep Seda Uyan, Ela Erdem Eralp, Ayse Ayzıt Kilinc, Ali Özdemir, Velat Şen, Erkan Cakir, Saniye Girit

**Affiliations:** ^1^ Division of Pediatric Pulmonology, Faculty of Medicine Medeniyet University Istanbul Turkey; ^2^ Division of Pediatric Pulmonology, Faculty of Medicine Marmara University Istanbul Turkiye; ^3^ Division of Pediatric Pulmonology, Cerrahpasa Faculty of Medicine Istanbul University Istanbul Turkiye; ^4^ Division of Pediatric Pulmonology Eskişehir City Hospital Eskişehir Turkiye; ^5^ Division of Pediatric Pulmonology, Faculty of Medicine Medipol University Istanbul Turkiye; ^6^ Division of Pediatric Pulmonology, Faculty of Medicine Koc University Istanbul Turkiye; ^7^ Division of Pediatric Pulmonology Mersin City Education and Training Hospital Mersin Turkiye; ^8^ Division of Pediatric Pulmonology, Faculty of Medicine Dicle University Diyarbakır Turkiye; ^9^ Division of Pediatric Pulmonology, Faculty of Medicine Istinye University Istanbul Turkiye

**Keywords:** long‐term outcomes, necrotizing pneumonia, parapneumonic effusion, radiologic sequelae, spirometry

## Abstract

**Background:**

Complications such as parapneumonic effusion (PPE) and necrotizing pneumonia (NP) can be noted in 3% of patients with community‐acquired pneumonia and may cause functional lung damage.

**Objective:**

We aimed to investigate the short‐ and long‐term effects of PPE and NP on lung function and the impact of treatment modalities and radiological sequelae on results.

**Material and Methods:**

This multicenter retrospective study includes children aged 0–18 years hospitalized for PPE and NP after community‐acquired pneumonia. Demographic, clinical, radiological, and spirometry findings were collected during diagnosis and follow‐up.

**Results:**

Of 123 children (62 female, median age 57 [interquartile range 71.5] months), 78 were diagnosed with NP and 45 with PPE. According to the defined periods, spirometric evaluation was performed in the first 3 months in 23 patients, between the 3rd and 6th months in 27 patients, and between the 6th and 9th months in 37 patients. At 3 months post‐discharge, abnormal spirometry (18.18% restrictive, 36.36% combined spirometry) was observed with a rate of 54.54% in NP, and with a rate of 25.0% (8.33% restrictive, 16.66% combined spirometry) in PPE. At 6‐9 months, normal spirometry was observed with a rate of 87% in both groups. FVC% values increased over time in both the NP and PPE groups; however, statistically significant improvement was observed only in the PPE group. In this group, FVC% was significantly higher in the patients who received antibiotics with chest tube and/or fibrinolytic therapy (*p* = 0.022). Furthermore, those without radiological sequelae had significantly higher FVC% values compared to those with sequelae (*p* = 0.023) in the PPE group.

**Conclusion:**

Radiological sequelae and restrictive spirometric patterns were initially more common in NP compared to PPE. However, spirometry indicated significant improvement in both groups by the end of the 9‐month follow‐up period.

## 1 Introduction

Parapneumonic effusion (PPE), necrotizing pneumonia (NP), and lung abscess are complications that occur in approximately 3% of pediatric cases of community‐acquired pneumonia (CAP) [1, 2]. These complications can extend the length of hospitalization and, in rare cases, may result in mortality [2, 3, 4].

NP is most commonly observed in children aged 2–5 years [3, 4, 5, 6]. Retrospective studies analyzing the incidence of NP have shown an increasing trend over the last two decades [4, 7, 8]. NP is a relatively rare but severe form of CAP, characterized by liquefaction and cavitation of the lung parenchyma, sometimes accompanied by impaired perfusion, usually complicated by PPE/empyema [3, 4, 5, 6]. For these reasons, NP is expected to present with a more severe clinical course, to have a delayed recovery compared with isolated PPE, and to show more significant and longer‐term consequences of lung injury.

The duration of radiological sequelae and lung function tests (LFTs) measurements have frequently been researched to investigate the structural and functional damage caused by CAP and its complications. Research on PPE (2%–12%), the most common complication of CAP, has continued for several decades. Interest in potential long‐term lung injury associated with NP, a rarer complication of CAP (3%–7%), has increased over the last decade [7]. Early retrospective studies with small cohorts examined the impact of PPE on LFTs. Still, recent prospective studies with larger sample sizes have concluded that PPE does not cause significant long‐term impairments in lung function [9, 10, 11]. The first study on long‐term sequelae of NP was published in 2021 [4]. LFTs and symptoms were assessed approximately 8.5 years after the diagnosis, and the long‐term clinical outcomes were generally favorable [4]. Lung parenchyma in patients with NP is expected to return to normal within a few months, but a limited number of patients may develop residual functional impairments such as small airway disease, bronchiectasis, and persistent reductions in lung capacity [12].

This study aimed to determine the short‐ and long‐term effects of isolated PPE (without NP) and NP on lung function, as assessed by spirometry at various recovery periods. Additionally, we planned to investigate the influence of treatment modalities and radiological sequelae on spirometry results.

## 2 Materials and Methods

### 2.1 Patients

**FIGURE 1:**
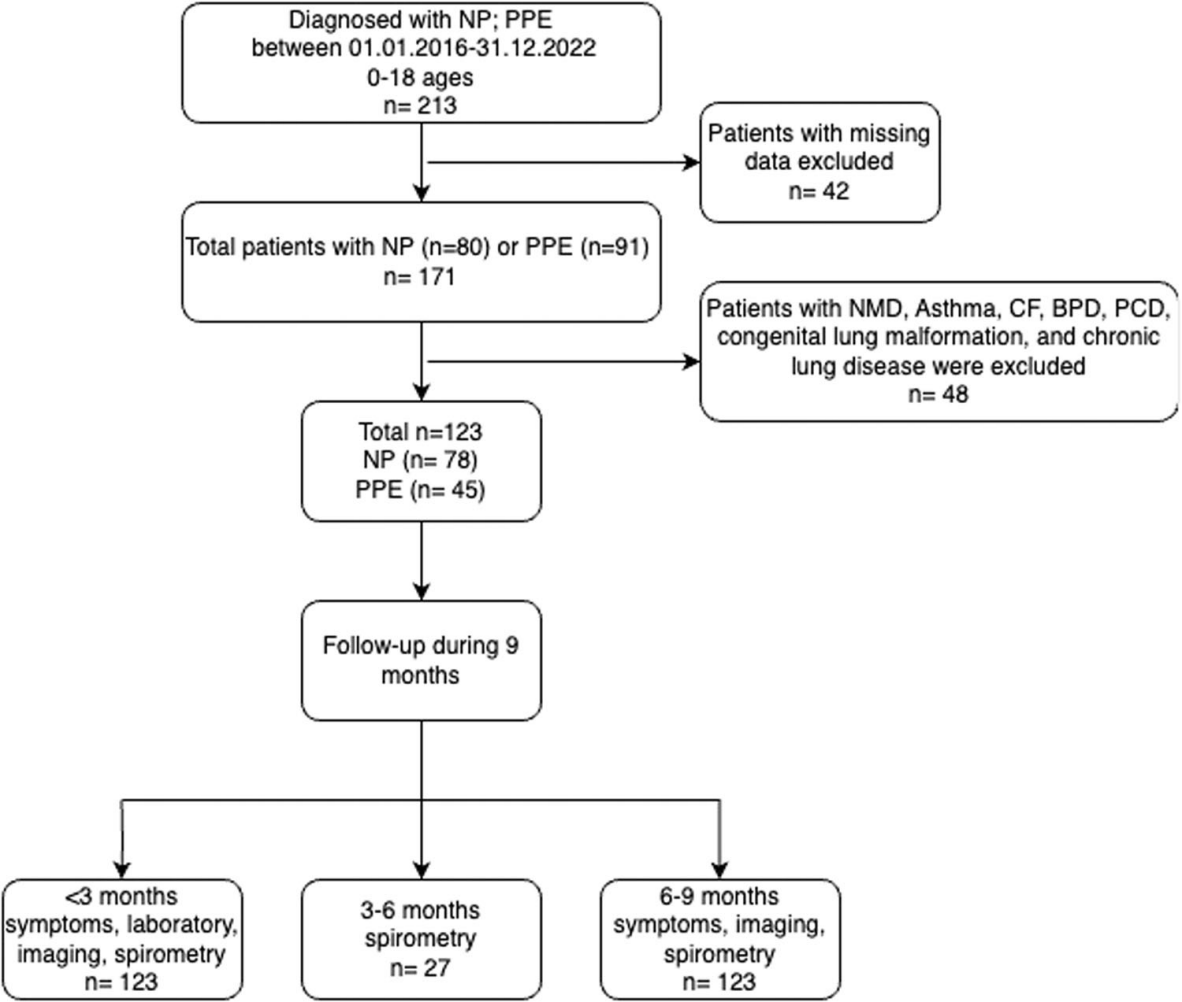
Flow diagram. BPD, bronchopulmonary disease; CF, cystic fibrosis; NMD, neuromuscular diseases; NP, necrotizing pneumonia; PPE, parapneumonic effusion; PCD, primary ciliary dyskinesia.

Our study was designed as a multicenter, retrospective cohort study. The Pediatric Pulmonology departments of eight tertiary care hospitals were included. Files of patients diagnosed with NP or isolated PPE who were hospitalized between January 1, 2016, and December 31, 2022, and who were under 18 years of age were reviewed, and patients with missing data were excluded (Figure 1).

Demographic information, clinical findings, laboratory and radiological imaging results, treatment modalities, and lengths of hospital stay were collected from the national online health database (https://enabiz.gov.tr), the hospital information management system, and patient records. Only cases of community‐acquired pneumonia (CAP) were included in the analysis. Patients with chronic respiratory diseases were excluded to prevent measurement bias (Figure 1). All children in the study were previously healthy and immunocompetent. The treatment model was divided into three main groups: antibiotics (AB) alone, AB and chest tube drainage (CTD), and AB, CTD, and fibrinolytic therapy. Post‐discharge follow‐up data of the patients were evaluated by dividing them into periods defined as < 3 months, 3–6 months, and 6–9 months (Figure 1).

This study was approved by the Research Ethics Committee of the Medeniyet University School of Medicine (approval number: 2023/0763). Written informed consent was obtained from the participants' parents, and appropriate participants received age‐appropriate assent after eligible patients were determined.

### 2.2 Radiology Imaging

Radiological data were evaluated through chest X‐rays, ultrasound (US), and chest CT scans to detect possible PPE, NP, air leak, or radiological sequelae. Patients with effusions < 10 mm were excluded, and loculations were recorded. Radiographic findings of NP include destruction of the lung parenchyma, loss of contrast enhancement, and the development of multiple thin‐walled ( < 2 mm) fluid‐ or air‐filled cavities [6]. Repeat radiological imaging, follow‐up chest X‐rays, US, or CT scans were performed if other radiographic abnormalities persisted [6]. Pneumothorax, pneumomediastinum, and bronchopulmonary fistula (BPF) were defined as air leak complications. As the patients were to be observed until their recovery was completed and chest radiographs returned to normal, radiological imaging conducted at 6–9 month follow‐up was reviewed. Atelectasis, BPF, bronchiectasis, and pleural thickening identified during the 6‐9‐month period were categorized as radiological sequelae.

### 2.3 Spirometry

Spirometry was performed on patients over 4 years old who could perform it. Spirometry measurements were conducted in the respiratory function laboratories of each center's pediatric pulmonology department by a single qualified lung function technician following the guidelines of the American Thoracic Society (ATS) and the European Respiratory Society (ERS) [13].

The following parameters were measured: forced expiratory volume in the first second (FEV_1_), forced vital capacity (FVC), and FEV_1_/FVC ratio. All spirometry measurements performed during the asymptomatic period were expressed as a percentage of the predicted normal values automatically calculated. Values greater than 80% were considered normal [14, 15, 16]. Also, we used the relevant prediction equations to calculate the lower limits of normal (LLN). We defined them as normal above the 5th percentile (z > −1.64) [17, 18]. The spirometry data were grouped into periods less than 3 months, 3–6 months, and 6–9 months post‐discharge.

The average value for each parameter was used if a patient underwent multiple spirometric evaluations within the same period. Obstructive pattern was defined as FEV_1_/FVC below 80% or LLN and FEV_1_ below 80% or LLN. Restrictive pattern was described as FEV_1_/FVC at or above 80% or normal LLN and FVC below 80% or LLN. Combined pattern was described as FEV_1_/FVC below 80% or LLN, low FEV_1_(% or LLN), and low FVC (% or LLN). Normal spirometry was defined as FEV_1_/FVC and FVC with normal LLN or above 80%.

A comparative analysis was conducted between the treatment model and radiological sequelae in the NP and PPE groups to ascertain which factor was associated with spirometric changes.

### 2.4 Statistics

Statistical analyses were performed using the SPSS software (IBM SPSS Statistics 27). The distribution of the variables was examined using the Shapiro–Wilk normality test. We expressed the data with normal or abnormal distribution as mean ± standard deviation (SD), median, and interquartile range (IQR), or absolute frequency and percentage. For comparing parametric variables, the “Independent Sample *t*‐test” was used for two independent groups, the “ANOVA” test was used for three or more independent groups, and the “Repeated Measures” test was used for three or more dependent groups. For comparing non‐parametric variables, the “Mann‐Whitney U” test was used for two independent groups, the “Kruskal‐Wallis H” test was used for three or more independent groups, and the “Friedman” test was used for three or more dependent groups. To examine the relationships between two categorical variables, Pearson's χ2 cross‐tables were used. For the paired statistical analysis, ANOVA was performed to compare the three time periods within both NP and PPE groups. ANOVA was used for normally distributed data; the Friedman test was applied for non‐parametric repeated measures. When statistically significant results were obtained, pairwise comparisons were conducted between the periods. A probability level lower than 0.05 was considered to assess statistical significance.

## 3 Results

We identified 213 patients diagnosed with NP and/or PPE as complications of CAP. As presented in Figure 1, 123 patients were included in the final analysis, of whom 78 were diagnosed with NP and 45 were diagnosed with PPE after excluding patients with missing data and with additional chronic lung diseases.

Table 1 lists demographic and clinical features. In the study population, the incidence of air leak as a complication was 20.32% (25/123), with a rate of 23.07% (18/78) in the NP group and with a rate of 15.55% (7/45) in the PPE group (*p* = 0.488). PPE/empyema was observed in 78.20% (*n* = 61) of the patients in the NP group.

**TABLE 1:** Demographic and clinical findings of the study population.

	Total	NP (*n* = 78)	PPE (*n* = 45)	*p*
Age, months, median, (IQR)	57 (71.5)	48.0 (73.3)	76.0 (66.0)	**0**.**007**
Female, n (%)	62 (50.4)	39 (50)	23 (51.1)	0.906
BMI (z‐score), median, (IQR)	0.05 (1.73)	0.18 (2.0)	0.02 (1.5)	0.537
Fever, days, median, (IQR)	4 (3)	4.0 (3.0)	3.0 (4.0)	0.179
Cough, days, median, (IQR)	6 (4.75)	5 (4)	5 (8)	0.667
Tachypnea, days, median, (IQR)	2 (2)	1 (2)	1 (2.5)	0.765
CRP (mg/dL), median, (IQR)	72 (206.0)	58.9 (189.9)	105.8 (225.0)	0.960
Length of hospital stay (days), median, (IQR)	25 (15.5)	28.0 (12.5)	21.0 (12.5)	**< 0**.**001**
Air leak complication, n (%)				
No	98 (79.6)	61 (78.2)	37 (82.2)	0.488
Yes	25 (20.3)	18 (23.1)	7 (15.55)	
Pneumothorax	24	17	7	
Px + Pm	4	3	1	
Px + BPF	3	3	—	
Pneumomediastinum	1	1	—	
OS and/or RS, n (%)				
No	55 (44.7)	30 (38.5)	25 (55.6)	0.099
Yes	68 (55.2)	48 (61.5)	20 (44.4)	
O_2_ via a mask	35 (28.4)	22 (28.2)	13 (28.8)	
HFNC	10 (8.1)	7 (8.9)	3 (6.6)	
NIMV	18 (14.6)	15 (19.2)	3 (6.6)	
IMV	5 (4.0)	4 (5.1)	1 (2.2)	
Treatment modalities, n (%)				
AB	41 (33.3)	26 (33.3)	15 (33.3)	
AB + CTD	46 (37.3)	30 (38.5)	16 (35.6)	0.929
AB + CTD + F	36 (29.2)	22 (28.2)	14 (31.1)	

*Note:* The bold values are indicate statistically significant *p* < 0.05.

Abbreviations: AB, antibiotics; BMI, body‐mass index; BPF, bronchopleural fistula; CRP, C‐reactive protein; CTD, chest tube drainage; F, fibrinolysis; HFNC, high‐flow nasal canula; IMV, invasive mechanical ventilation; IQR, interquartile range; NIMV, noninvasive mechanical ventilation; NP, necrotizing pneumonia; O_2_, oxygen; OS, oxygen supplementation; pneumomediatinum; PPE, parapneumonic effusion; Px + Pm, pneumothorax + RS, respiratory support.

During hospitalization, 55.28% of all patients (*n* = 68) required oxygen and/or respiratory support, with rates of 61.5% in the NP group and 44.4% in the PPE group (Table 1). A statistical analysis revealed no significant difference between the two groups (*p* > 0.05) (Table 1).

During the hospitalization, all children received parenteral AB (Table 1). Patients diagnosed with NP were hospitalized for a more extended period than those in the PPE group (*p* < 0.001) (Table 1). Consequently, radiological evaluations conducted at the 6–9 months revealed that 56.41% (*n* = 44) of the patients with NP and 40.0% (*n* = 18) of the patients with PPE exhibited radiological sequelae. The rate of radiological sequelae was found to be similar in both groups (*p* = 0.080). In the NP group, atelectasis, bronchiectasis, pleural thickening, and BPF were detected with rates of 26.92% (*n* = 21), 3.84% (*n* = 3), 41.02% (*n* = 32), and 2.56% BPF (*n* = 2), respectively. In the PPE group, atelectasis, bronchiectasis, and pleural thickening were detected with rates of 28.88% (*n* = 13), 6.66% (*n* = 3), and 22.22% (*n* = 10), respectively.

According to the defined periods, spirometric evaluation was performed in the first 3 months in 23 patients, between the 3rd and 6th months in 27 patients, and between the 6th and 9th months in 37 patients. In the first 3 months after discharge, 13.04% (3/23) of the patients exhibited a restrictive pattern, 26.08% (6/23) demonstrated a combined pattern, and 60.8% (14/23) showed a normal spirometry pattern. In the 6–9 month period, 13.51% (5/37) of the patients exhibited a restrictive pattern, and 86.49% (32/37) displayed normal spirometry. Remarkably, no obstructive pattern was detected in any period (Table 2, Supporting file). No statistically significant differences were observed in spirometric parameters between the NP and PPE groups (Table 3). In the NP group, spirometry results were available for all three time intervals in six patients. The only parameter that showed a statistically significant increase was the FVC% value (*p* = 0.048) (Table 4). The FVC% values of the NP group were similar during the < 3 months and 3–6 month period, but showed a statistically significant improvement in the 6–9 month period compared to the < 3 month period (*p* = 0.011). In the PPE group, spirometry results were available for all three time intervals in five patients. The parameters that showed a statistically significant increase were the FVC% and FEV_1_/FVC values (*p* = 0.049 and *p* = 0.16, respectively) (Table 4). The PPE group demonstrated a marked increase in FVC% values during the second interval compared to the first interval (*p* = 0.012). Additionally, FEV_1_/FVC demonstrated significant improvements between < 3 months and 3–6 months, as well as between < 3 months and 6–9 months (*p* = 0.002, *p* < 0.001, respectively) (Table 4).

**TABLE 2:** Spirometry of NP/PPE groups at three different time periods.

	NP (*n* = 78)	PPE (*n* = 45)
< 3 months		
Number of spirometry	*n* = 11	*n* = 12
Post‐discharge time, days, median (IQR)	48 (41.5)	88 (26.2)
Normal spirometery %(n)	45.4 (*n* = 5)	75.0 (9)
Abnormal spirometry %(n)	54.5 (*n* = 6)	25.0 (*n* = 3)
Restrictive %(n)	18.1 (*n* = 2)	8.3 (*n* = 1)
Obstructive %(n)	0	0
Combined %(n)	36.3 (*n* = 4)	16.6 (2)
3–6 months		
Number of spirometry	*n* = 13	*n* = 14
Post‐discharge time, days, median (IQR)	166 (43)	120 (43.7)
Normal spirometry % (n)	30.7 (*n* = 4)	57.1 (*n* = 8)
Abnormal spirometry % (n)	69.2 (*n* = 9)	42.8 (*n* = 6)
Restrictive % (n)	46.1 (*n* = 6)	28.5 (*n* = 4)
Obstructive % (n)	0	0
Combined % (n)	23.0 (*n* = 3)	14.2 (*n* = 2)
6–9 months		
Number of spirometry	*n* = 22	*n* = 15
Post‐discharge time, days, median (IQR)	251 (42)	236 (48)
Normal spirometry % (n)	86.3 (*n* = 19)	86.6 (*n* = 13)
Abnormal spirometry % (n)	13.6 (*n* = 3)	13.3 (*n* = 2)
Restrictive % (n)	13.6 (*n* = 3)	13.3 (*n* = 2)
Obstructive % (n)	0	0
Combined % (n)	0	0

Abbreviations: IQR, interquartile range; NP, necrotizing pneumonia; PPE, parapneumonic effusion.

**TABLE 3:** Comparison of spirometry parameters of NP/PPE groups at three different time periods during follow‐up.

	NP (*n* = 78)	PPE (*n* = 45)	*p*
**FEV**_**1**_ **(%), mean ** ± **SD (n)**			
< 3 months	84.4 ± 18,2 (11)	88.0 ± 22.5 (12)	0.673
3–6 months	79.5 ± 21,5 (13)	90.1 ± 11.3 (14)	0.118
6–9 months	88.1 ± 18,6 (22)	96.4 ± 11.9 (15)	0.097
**FEV**_**1**_ **(z‐score), median (IQR) (n)**			
< 3 months	−0.8 (1,9) (11)	−0.8 (1.5) (12)	0.400
3–6 months	−0.4 (2,0) (13)	−0.6 (1.1) (14)	0.964
6–9 months	−0.9 (1,3) (22)	−0.4 (1.1) (15)	0.188
**FVC (%), mean ** ± **SD (n)**			
< 3 months	74.1 ± 12.1 (11)	**82.0 ± 16.6 (12)**	**0.157**
3–6 months	73.1 ± 19.5 (13)	**85.0 ± 10.9 (14)**	0.113
6–9 months	84.7 ± 20.8 (22)	89.5 ± 9.7 (15)	0.352
**FVC (z‐score), median (IQR) (n)**			
< 3 months	−1.2 (2.5) (11)	**−1.1 (2.0) (12)**	0.587
3–6 months	−0.8 (2,5) (13)	−1.0 (2.1) (14)	0.357
6–9 months	−1.1 (2,0) (22)	−0.7 (1.1) (15)	0.712
**FEV**_**1**_ **/FVC (%), mean ** ± **SD (n)**			
< 3 months	106.6 ± 7,7 (11)	**103.4 ± 12.5 (12)**	0.816
3–6 months	104.3 ± 6.6 (13)	105.0 ± 10.8 (14)	0.858
6–9 months	104.3 ± 8.0 (22)	106.6 ± 6.2 (15)	0.580
**FEV**_**1**_ **/FVC (z‐score), median (IQR) (n)**			
< 3 months	0.8 (1.9) (11)	1.0 (2.0) (12)	0.221
3–6 months	0.14 (1.2) (13)	1.2 (2.2) (14)	0.071
6–9 months	0.6 (1.9) (22)	0.9 (2.3) (15)	0.201

Abbreviations: FEV_1_, forced expiratory volume in the first second; FVC, forced vital capacity; IQR, interquartile range; NP, necrotizing pneumonia; PPE, parapneumonic effusion; SD, standard deviation.

**TABLE 4:** Paired comparison of spirometry parameters over time within NP and PPE groups.

	NP (*n* = 6)	*p*	PPE (*n* = 5)	*p*
	Median (min‐max)		Median (min‐max)	
FEV_1_ (%)				
**<** 3 months	86.5 (47–110)		89.0 (71–104)	
3–6 months	81.5 (51–110)	0.568	98.0 (86–102)	0.486
6–9 months	89.5 (56–98)		93.0 (79–104)	
FEV_1_ (z‐score)				
< 3 months	−0.77 (−4.43 to −0.32)		−0.84 (−2.08 to 0.48)	
3–6 months	−0.71 (−4.71 to −1.40)	0.568	−0.12 (−0.86 to 0.23)	1.000
6–9 months	−1.04 (−2.74 to 1.40)		−0.59 (−1.10 to 0.42)	
FVC (%)				
< 3 months ^(1)^	73.5 (49–85)		83.0 (63–94)	
3–6 months ^(2)^	74.5 (54–82)	**0.048**	88.0 (74–101)	**0.049**
6–9 months ^(3)^	81.5 (52–93)	* **(0.011)**	87.0 (74–105)	** **(0.012)**
FVC (z‐score)				
< 3 months	−1.20 (−4.43 to −0.32)		−1.30 (−2.87 to −0.42)	
3–6 months	−0.92 (−3.95 to 1.26)	0.119	−0.38 (−1.05 to 0.17)	0.247
6–9 months	−1.01 (−3.32 to 1.26)		−0.65 (−1.70 to 0.46)	
FEV_1_ /FVC (%)				
< 3 months	110 (96–118)		109 (104–114)	
3–6 months	108 (93–116)	0.331	104 (100–111)	0.504
6–9 months	108 (92–118)		103 (98–110)	
FEV_1_ /FVC (z‐score)				
< 3 months ^(1)^	0.53 (−0.58 to 1.97)		2.28 (0.76–3.47)	**0.016**
3–6 months ^(2)^	0.16 (−0.90 to 0.86)	0.200	0.03 (−1.42 to 2.19)	*** **(*p*** = **0.002)**
6–9 months ^(3)^	0.31 (−1.19 to 1.79)		0.61 (−0.29 to 2.19)	**** **(*p*** < **0.001)**

*Note:* The bold values are indicate statistically significant *p* < 0.05.

* (0.011): a statistically significant difference between < 3 month and 6–9 month period.

** (0.012): a statistically significant difference between < 3 month and 3–6 month period.

*** (*p* = 0.002): a statistically significant difference between < 3 month and 3–6 month period.

**** (*p* < 0.001): a statistically significant difference between < 3 month and 6–9 month period.

The current study detected the factors that may affect the change in FVC%, particularly regarding treatment modality, radiological sequelae, and CRP. The increase in FVC% in the PPE group was associated with a change in treatment modality (*p* = 0.022) (Table 4). In the PPE group, this revealed that the FVC% value of the patients who received the AB + CTD+fibrinolysis treatment model during the initial 3‐month period exceeded that of the patients who received the AB+ chest tube treatment model (*p* = 0.022) (Table 5). In the NP group, the increase in FVC% value was not associated with the treatment model (*p* = 0.647).

**TABLE 5:** Relation of FVC% change to treatment modality and radiological sequelae.

	< 3 months	3–6 months	6–9 months
	mean ± SD	mean ± SD	mean ± SD
**NP group**			
AB	68.6 ± 11.2	71.0 ± 22.9	78.8 ± 25.0
AB + CTD	72.6 ± 16.2	80.0 ± 20.3	91.4 ± 18.0
AB + CTD + F	78.6 ± 3.0	99.5 ± 14.8	91.5 ± 12.9
***p***	0.647	0.310	0.377
Radiological sequelae (‐)	74.8 ± 14.5	63.8 ± 22.2	73.3 ± 18.9
Radiological sequelae (+)	71.8 ± 11.0	80.6 ± 15.6	93.6 ± 1.2
***p***	0.710	0.381	0.067
**PPE group**			
AB ^(1)^	82.8 ± 9.8	93.8 ± 6.9	94.8 ± 10.9
AB + CTD ^(2)^	64.0 ± 15.8	85.4 ± 17.0	90.2 ± 4.1
AB + CTD + F ^(3)^	98.3 ± 15.3	90.6 ± 5.5	103.4 ± 15.2
***p***	**0**.**022 (2‐3)**	0.504	0.250
Radiological sequelae (‐)	84.7 ± 12.9	87.6 ± 10.2	89.7 ± 11.0
Radiological sequelae (+)	76.0 ± 24.3	77.6 ± 9.9	89.0 ± 6.3
*p*	0.643	**0**.**023**	0.904

*Note:* The bold values are indicate statistically significant *p* < 0.05.

*Note:* **(2‐3):** a statistically significant difference between AB + CTD and AB + CTD + F.

Abbreviations: AB, antibiotics; CTD, chest tube drainage; F, fibrinolysis; NP, necrotizing pneumonia; PPE, parapneumonic effusion; SD, standard deviation.

In the PPE group, the second evaluation interval (3–6 months) revealed decreased FVC% value in the patients who had radiological sequelae (*p* = 0.023). Conversely, the NP group exhibited no correlation between FVC% change and radiological sequelae at any evaluation intervals (*p* > 0.05) (Table 5). Following a comprehensive evaluation of the entire patient cohort (*n* = 123), a correlation was identified between the applied treatment model and the occurrence of radiological sequelae. The rate of radiological sequelae was higher in the patients who received the AB + CTD+fibrinolysis treatment compared to those who received AB alone or AB + CTD (*p* = 0.021). The rate of radiological sequelae was higher in those who required oxygen supplementation and/or respiratory support (*p* = 0.038). The CRP value (median 114.5 mg/dL (IQR 223.1)) of the patients with radiological sequelae was statistically higher compared to the CRP value (median 22.2 mg/dL [IQR 191.9]) of the patients without sequelae (*p* = 0.004) (Table 6).

**TABLE 6:** Analysis of clinical features that may be associated with radiological sequelae.

	Radiological sequelae		
	No (*n* = 61)	Yes (*n* = 62)	*p*
Gender			
Male, n (%)	32 (52.5)	29 (46.8)	0.528
Female, n (%)	29 (47.5)	33 (53.2)	
Air leak complications			
No, n (%)	50 (82.0)	47 (75.8)	0.403
Yes, n (%)	11 (18.0)	15 (24.2)	
Treatment modalities			
AB	25 (41.0)	16 (25.8)	
AB + CTD	25 (41.0)	21 (33.9)	
AB + CTD + F	11 (18.0)	25 (40.3)	**0**.**021**
OS and/or RS			
No	33 (54.1)	22 (35.5)	
Yes	28 (45.9)	40 (64.5)	**0**.**038**
CRP, median (IQR)	22.2 (191.9)	114.5 (223.1)	**0**.**004**

*Note:* The bold values are indicate statistically significant *p* < 0.05.

Abbreviations: AB, antibiotics; CRP, C‐reactive protein; CTD, chest tube drainage; F, fibrinolysis; IQR, interquartile range; OS, oxygen supplementation; RS, respiratory supplementation.

Persistent symptoms for 6–9 months were as follows: cough 22% (17/78) and sputum 13% (10/78) in the NP group; cough 15% (7/45), wheeze 9% (4/45), and sputum 9% (4/45) in the PPE group, with no statistically significant difference (*p* > 0.05). There were no deaths in either group during the treatment or follow‐up periods.

## 4 Discussion

We analyzed the long‐term follow‐up of NP and PPE. We found restrictive patterns were the most common short‐term spirometric abnormalities in both groups, particularly in NP. During follow‐up, the NP group exhibited a more pronounced improvement in spirometric parameters compared to the PPE group. However, spirometric measurements in both groups approached normal values at long‐term follow‐up (6–9 months). The rate of abnormal spirometry decreased from 55% to 13% in the NP group and from 25% to 13% in the PPE group.

Previously healthy children are expected to recover almost completely clinically and radiologically within 6‐9 months following complicated CAP. Still, close follow‐up is necessary after discharge due to the risk of complications such as pneumatocele and NP [2]. Patients with PPE should be monitored until complete recovery and normalization of chest radiographs, which typically occurs within 3‐6 months [19]. It is predicted that this period may take longer in NP [2]. The long‐term follow‐up of patients with PPE has been investigated for many years, while studies on the long‐term outcome of patients with NP have gained momentum in recent years [4, 11, 20, 21].

In the literature, PPE/empyema accompanies NP in 63%–100% of cases. In our study, PPE/empyema was observed in 78.20% of the patients in the NP group. Other frequently observed complications include pneumothorax, detected in 16–25% of cases on radiographic imaging, and BPF, identified in 16%–30% of cases based on persistent ( > 24 h) air leakage on CT [7, 16, 22, 23, 24]. Air leak complications were present in 20% of the NP group (17 cases of pneumothorax, four cases of pneumomediastinum, and three cases of BPF). In the PPE group, air leak complications were observed in 15% of cases, consisting of seven cases of pneumothorax and one case of pneumomediastinum.

In the last 6–9 months of follow‐up, radiological sequelae were observed in 56% of the NP and 40% of the PPE groups. In the NP group, atelectasis, bronchiectasis, pleural thickening, and BPF were detected with rates of 27%, 4%, 41%, and 2.5%, respectively. In the PPE group, atelectasis, bronchiectasis, and pleural thickening were detected with rates of 29%, 6%, and 22%, respectively. Atelectasis and bronchiectasis were similar between the groups, whereas pleural thickening was more common in the NP group. Krenke et al. demonstrated that all NP patients experienced complete or near‐complete resolution of residual pulmonary and pleural lesions within 5 months [7]. Satish et al. demonstrated that all radiological findings resolved within 2–16 months following empyema and found no association between long‐term radiological sequelae and LFTs results [19]. deBenedict et al. observed that radiological findings such as consolidation, atelectasis, pneumatocele, pneumothorax, and pleural thickening persisted in patients at discharge, most of them resolved within 2–13 months, and only 10.25% of the patients had long‐term sequelae [9]. Most studies have evaluated radiological sequelae at 12 months or longer. The high rate of radiological sequelae observed in our study in the 6–9 month period may be due to early evaluation. Chest X‐rays may often be insufficient for follow‐up; therefore, it is essential to use more sensitive methods such as ultrasound and, when necessary, CT [25]. Additionally, it is essential to note that the rate of use of better diagnostic procedures such as USG and CT may be lower in reports in the past decade. Various studies have reported a higher tendency for pleural thickening in patients with empyema [26].

We examined the factors influencing radiological sequelae. We determined that sequelae were more common in patients receiving AB + CTD+fibrinolysis, requiring oxygen or respiratory support, and showing elevated CRP levels. Ramaslı et al. found that the delayed resolution of pleural thickening following PPE was associated with preadmission fever, low oxygen saturation, length of hospital stay, and fibrinolytic treatment. McKee et al. identified BPF in 33% of cases following empyema and demonstrated that the duration of pleural drainage, length of hospital stay, and surgical intervention influenced the rate of BPF [27]. Although we could not define empyema as a separate group within PPE, fibrinolytic therapy in approximately one‐third of both our NP and PPE groups supported the presence of empyema in these patients. The rate of fibrinolytic therapy was higher in patients who had radiological sequelae (40%) compared to in those who did not (18%). This observation may appear paradoxical; however, these findings suggest that pleural thickening could be more pronounced in severe cases necessitating fibrinolytic therapy or in NP cases associated with PPE. Similar to the literature, our results indicate that radiological sequelae are associated with more severe disease characteristics.

A restrictive pattern on spirometry may occur due to residual parenchymal or pleural disease in empyema, where the loculations bridging the parietal and visceral pleura have not entirely resolved, thus preventing full lung expansion [11]. Until recently, information on children with NP was mainly based on case reports and small case series, and no longitudinal study analyzed lung function [4]. In the first 3 months, we found abnormal spirometry in only 25% of the PPE group and in 55% of the NP group. This rate increased to 43% in the PPE group to 69% in the NP group in the 3–6 month period. In the 6–9 month period, the rate of spirometry abnormality decreased to 13% in both groups, and 87% were found to have normal spirometry results. In the PPE studies, restrictive pattern was observed with a ate of up to 91% in LFTs performed in 3 months post‐discharge [11]. Maffey et al. reported that 93% of patients exhibited a restrictive pattern at discharge following empyema. By the end of 6 months, however, only one case of restrictive pattern persisted, while the others returned to normal [28]. Göçmen et al. performed LFT in 25 of 72 children with empyema after a mean of 18 months, and all were evaluated as having normal patterns [29]. Bover‐Bouza et al. evaluated 24 patients with NP at the end of the 2nd year and reported that spirometric parameters were within normal limits [4]. Our findings indicate that further improvements may be achieved over time. In the NP group, the mean FVC percentage remained slightly below the lower limit during the first and second 3 months but showed a significant increase in the third 3 months. Meanwhile, FVC z‐scores were near normal values, with no significant variations observed.

In the current study, the mean FVC% value in the NP group was slightly lower compared to the PPE group. A significant rise in FVC% values was observed in the NP group between the second and third 3 months, while a continuous rise over time was observed in the PPE group. FVC z‐scores remained near normal levels, with no notable changes detected. We hypothesize that extensive parenchymal damage in NP may have had a more significant impact on lung expansion, leading to a delayed normalization of mean FVC values due to the prolonged recovery of parenchymal tissue. It is known that spirometry tends to normalize within a few months in PPE, regardless of the treatment modality [2, 28]. Redding et al. evaluated a total of 15 patients with and without CTD with empyema after 5 years and found no significant difference in spirometry results [30]. However, our study identified a correlation between FVC% values, treatment modality, and radiological sequelae. Higher FVC% values were observed in PPE patients who received fibrinolytic therapy and who had no radiological sequelae. This finding suggests fibrinolytic therapy may reduce restrictive lung impairment by decreasing loculation within the pleural space. In the NP group, neither the treatment modality nor the radiological sequelae indicated a statistically significant impact on spirometry. The lack of a substantial relationship in the NP group could be due to the NP's intrinsic nature, which makes spirometric outcomes less dependent on treatment type or sequelae. Similarly, Bover‐Bouza et al. found no significant difference in spirometry abnormalities in patients with NP when comparing patients with and without pleural drainage [4]. Frybova et al. found that 64% of 81 children with NP who underwent surgery or CTD had normal spirometric values at 12 months [20]. Specifically, spirometries were normal in 56% of those who underwent lung resection, 66% of those who underwent decortication, and 75% of those who underwent CTD [20]. The rate of spirometric improvement was higher in patients who underwent CTD but not surgical intervention such as resection or decortication. Considering our results and the literature, parenchymal injury caused by surgical procedures and NP, has been demonsrated to impact LFTs in addition to fibrinolytic therapy and CTD. Furthermore, the severity of pleural and parenchymal inflammation in NP or PPE, differences in the patient populations studied, and patient selection may contribute to variations in long‐term spirometric outcomes.

Our study has several limitations including primarily its retrospective nature, which may have led to gaps in the medical records. Empyema could not be analyzed separately due to insufficient pleural fluid data. Since the participants were previously healthy children, pre‐diagnosis spirometry values were unavailable. Lung function was assessed by spirometry only, as plethysmography was not available for all centers. Fewer patients than the total cohort had spirometry results, and this number varied between follow‐up periods. The strength of our study includes its contribution to the literature by comparing the long‐term outcomes of children with NP and PPE for the first time. Despite being retrospective, the follow‐up evaluations conducted in three distinct periods allowed for a better understanding of the changes in spirometric values.

## 5 Conclusion

In childhood, NP and PPE do not appear to cause long‐term impairment in spirometry; however, a restrictive pattern is the most common abnormality in the early period. Our findings suggest neither condition results in persistent deterioration of spirometry in the long term, highlighting the potential for pulmonary recovery with appropriate management and follow‐up.

## Author Contributions

**Sinem Can Oksay:** conceptualization (equal), data curation (equal), formal analysis (equal), investigation (equal), methodology (equal), project administration (equal), resources (equal), writing original draft (equal), writing review and editing. **Begüm Yörük:** data curation (equal), project administration (equal), methodology (equal), resources (equal), writing original draft (equal). **Şeyda Karabulut:** data curation (equal), project administration (equal), methodology (equal), writing original draft (equal). **Huseyin Arslan:** data curation (equal), project administration (equal), methodology (equal), software (equal). **Gülay Bilgin:** data curation (equal), writing original draft (equal), project administration (equal), methodology (equal), software (equal). **Fusun Unal:** data curation (equal), project administration(equal), software (equal). **Deniz Mavi Tortop:** data curation (equal), project administration(equal), methodology (equal), writing original draft (equal). **Ebru Kostereli:** data curation (equal), project administration (equal), software (equal), methodology (equal). **Zeynep Reyhan Onay:** data curation (equal), project administration(equal), methodology (equal), software (equal). **Yasemin Gokdemir:** data curation (equal), project administration (equal), software (equal). **Zeynep Seda Uyan:** data curation (equal), project administration (equal), methodology (equal), software (equal). **Ela Erdem Eralp:** data curation (equal), project administration (equal), software (equal). **Ayse Ayzıt Kilinc:** data curation (equal), project administration (equal), writing original draft (equal). **Ali Özdemir:** data curation (equal), project administration (equal), software (equal). **Velat Şen:** data curation (equal), project administration (equal), software (equal). **Erkan Cakır:** data curation (equal), project administration(equal), methodology (equal), software (equal). **Saniye Girit:** Conceptualization (lead); data curation (lead), formal analysis (lead), investigation (lead), methodology (lead), project administration (lead), resources (lead), supervision (lead), writing original draft (lead), writing review and editing (lead). All authors approved the final manuscript as submitted and agreed to be accountable for all aspects of the work.

## Ethics Statement

This study was approved by the Research Ethics Committee of the Medeniyet University School of Medicine (approval number: 2023/0763).

## Consent

Written informed consent was obtained from participants' parents, and appropriate participants received age‐appropriate assent. This study was presented as an oral presentation at the Pediatric Pulmonology Society's 6th Annual Congress in 2023. It was deemed worthy of the “Oral Presentation Third Award.”

## Conflicts of Interest

The authors declare no conflicts of interest.

## Supporting information

supporting File Long‐Term Outcomes of Necrotizing Pneumonia and Parapneumonic Effusion in Children.

## Data Availability

The data that support the findings of this study are available on request from the corresponding author. The data are not publicly available due to privacy or ethical restrictions.
