# Supplementary material for: Long‐Term Outcomes of Necrotizing Pneumonia and Parapneumonic Effusion in Children
Source: Pediatr Pulmonol. 2025 Aug 19;60(8):e71241. doi: 10.1002/ppul.71241 (PMC12363156; doi:10.1002/ppul.71241)
Supplement: Supplementary file 1 — supporting File Long‐Term Outcomes of Necrotizing Pneumonia and Parapneumonic Effusion in Children. [file PPUL-60-0-s001.docx]

**Supplementary File 1**. Changes in lung function test results according to periods.


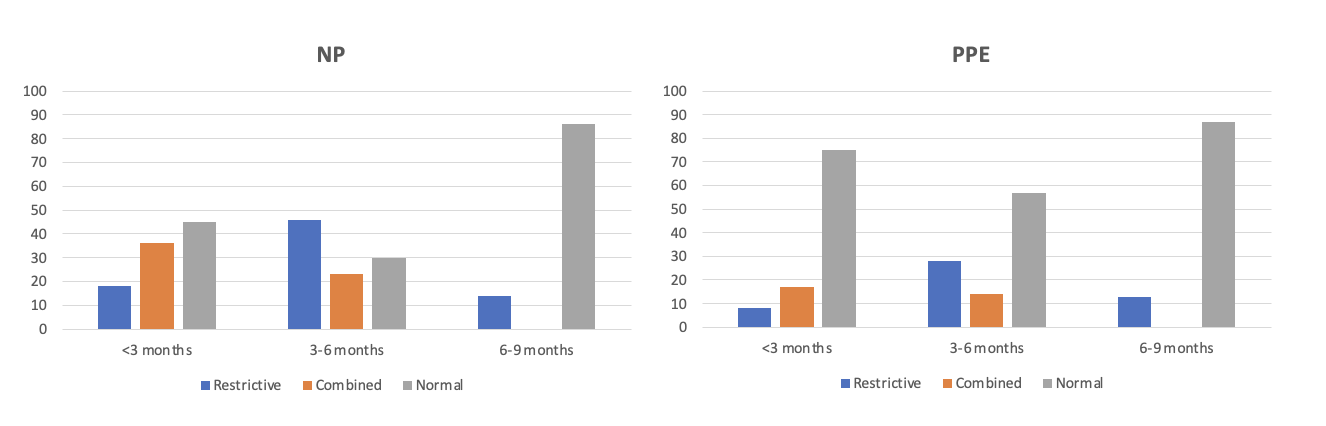


**NP:** necrotizing pneumonia, **PPE:** parapneumonic effusion

**Supplementary File 2.** Comparison of the current study with previous studies

|  | Current Study | Bover-Bouza et al.^4^ | Frybova et al.^20^ | Sawicki et al.^25^ | Cohen et al.^10^ | deBenedict et al.^9^ | Kohn et al.^11^ |
| --- | --- | --- | --- | --- | --- | --- | --- |
| Country | Turkiye | Spain | Czech Republic | USA | Canada | Italy | USA |
| Adjustment | Tertiary care centres; Pediatric pulmonology clinic | Tertiary care centre | Tertiary Referral Centre, which provides thoracic surgical service | Childrenn’s Hospital Boston | Tertiary care centre; | General hospital; Tertiary care  Pediatrichospital | Children’s hospital medical center for empyema |
| Design of the study | Retrospective cohort | Prospective | Prospective | Retrospective | Prospective | Prospective | Cross-sectional |
| Number of patients | 123 NP and isolated PPE | 24 NP | 81 NP | 80 NP | 82 empyema | 39 empyema | 36 empyema |
| Age at illness, median | 48 months for NP  76 months for PPE | 26 months | 32.5 months  Time of surgery | 3.6 years | 3.6 years | 4.6 years | 9.5 years |
| Intervention | AB: n=41  AB+CTD: n=46  AB+CTD+fibrinolysis: n=36 | Chest tube: n=18  CTD+fibrinolysis: n=11 | CTD: n=24  CTD+fibrinolysis: n=11  Decortication: n=10  Lung resection: n= 36 | CTD: n= 32  Thrombolysis: n=17  VATS: n=12  Partial lung resection: n=1  Decortication: n=3 | AB: n=31  CTD: n=11  CTD+fibrinolysis: n=40 | AB: n=39  CTD±fibrynolisis: n=20  VATS: n=19 | Thoracic surgery: n=21  VATS: n=14  Thoracotomy: n=7 |
| Length of hospital stay, days | 28 / 21 | 15 | 35 days for boys,  45 days for girls | 12 | 12 | 16 | 11.2 |
| Spirometry time | Post-discharge  <3 months, 3-6 months, 6-9 months | Median 8.75 years after NP episode | 43.3 months after surgery | Within a median of 6 months post-discharge | 3 post-discharge points as 1^st^, 6^th^, 12^th^ month | Postdischarge first visit, (1^st^ month) | Postdiascharge 2.4 years  (1week–8years) |
| Spirometry results | **<3 months: n=23**  Normal: 61%  Restrictive: 13%  Combined: 26%  **3-6 months: n=27**  Normal: 44%  Restrictive: 37%  Combined: 19%  **6-9 months: n=37**  Normal: 87%  Restrictive: 13% | All patients had normal spirometry | **n=54**  Normal: 65%  Restrictive: 11%  Obtructive 20%  Combined: 4% | **n=12**  Normal: 67%  Restrictive: 8%  Obstructive: 25% | **1^st^ month: n=20**  Normal: 65%  Abnormal: 35%  **6^th^ month: n=29**  Normal: 97%  Abnormal:3%  **12^th^ month: n=34**  Normal: 94%  Abnormal: 6% | **n= 17**  Normal: 29%  Abnormal:71%  (mainly mixed) | **<3 months: n=11**  Normal: 9%  Restrictive: 91%  **3-12 months: n=7**  Normal: 43%  Restrictive: 29%  Obstructive: 28%  **>12 months: n=26**  Normal: 66%  Restrictive: 19%  Obstructive: 15% |

**NP:** necrotizing pneumonia, **PPE:** parapneumonic effusion, **AB:** antibiotics, **CTD:** chest tube drainage, **VATS:** video assisted-thoracic surgery, **USA:** United States of America
